# The hemolytic and cytolytic activities of *Serratia marcescens *phospholipase A (PhlA) depend on lysophospholipid production by PhlA

**DOI:** 10.1186/1471-2180-9-261

**Published:** 2009-12-16

**Authors:** Ken Shimuta, Makoto Ohnishi, Sunao Iyoda, Naomasa Gotoh, Nobuo Koizumi, Haruo Watanabe

**Affiliations:** 1 Department of Bacteriology I, National Institute of Infectious Diseases, Toyama 1-23-1, Shinjuku-ku, Tokyo 162-8640, Japan; 2 Department of Microbiology and Infection Control Sciences, Kyoto Pharmaceutical University, Misasagi-Nakauchicho 5, Yamashinaku, Kyoto, 607-8414, Japan

## Abstract

**Background:**

*Serratia marcescens *is a gram-negative bacterium and often causes nosocomial infections. There have been few studies of the virulence factors of this bacterium. The only *S. marcescens *hemolytic and cytotoxic factor reported, thus far, is the hemolysin ShlA.

**Results:**

An *S. marcescens shl*AB deletion mutant was constructed and shown to have no contact hemolytic activity. However, the deletion mutant retained hemolytic activity on human blood agar plates, indicating the presence of another *S. marcescens *hemolytic factor. Functional cloning of *S. marcescens *identified a phospholipase A (PhlA) with hemolytic activity on human blood agar plates. A *phl*AB deletion mutant lost hemolytic activity on human blood agar plates. Purified recombinant PhlA hydrolyzed several types of phospholipids and exhibited phospholipase A1 (PLA1), but not phospholipase A2 (PLA2), activity. The cytotoxic and hemolytic activities of PhlA both required phospholipids as substrates.

**Conclusion:**

We have shown that the *S. marcescens phlA *gene produces hemolysis on human blood agar plates. PhlA induces destabilization of target cell membranes in the presence of phospholipids. Our results indicated that the lysophospholipids produced by PhlA affected cell membranes resulting in hemolysis and cell death.

## Background

*Serratia marcescens *is widely distributed in natural environments and has emerged in the last two decades as an important nosocomial pathogen, mainly in immunocompromised patients [1,2]. Although *S. marcescens *pathogenicity is poorly understood, its extracellular secreted enzymes, including several types of proteases, are candidates for virulence factors [2]. Other factors (e.g., fimbria for adhesion, lipopolysaccharide (LPS), and ShlA hemolysin) have also been suggested as virulence factors [2,3].

Hemolysins are produced by various pathogenic bacteria and have been proposed to be responsible for their pathogenesis [4-6]. These hemolysins, including *S. marcescens *ShlA, also have cytolytic activity [7]. One type of hemolysin/cytolysin is a group of pore-forming toxins. This type of toxin typically forms a homo-oligomer integrated into its target cell membrane, thereby changing the cell permeability and leading to cell death. ShlA has been shown to increase cell membrane permeability, but not to form an oligomer [3]. Another type of hemolysin has phospholipase C (PLC) activity. The α-toxin produced by *Clostridium perfringens *is the most thoroughly investigated PLC, but the molecular mechanism for its disruption of red blood cells (RBC) is not fully understood [8]. The pathogenic effects of other types of phospholipases, such as phospholipase A (PLA), have been studied in various bacteria, including *Helicobacter pylori *(PldA) [9], *Legionella pneumophila *(PlaA) [10], *Campylobacter coli *(PldA) [11], and *Yersinia enterocolitica *(YplA) [12]. Two extracellular PLAs, PhlA and PlaA, have been described previously in *Serratia *species [13,14]. PlaA is produced in *Serratia *sp. strain MK1 isolated from Korean soil [14]. The amino acid sequence of PlaA was found to have significant similarity (80%) to PhlA from *S. marcescens *MG1, which was originally classified as *S. liquefaciens *[13-15]. However, the cytotoxic and hemolytic activities of these enzymes have remained unclear, and the importance of PLA in bacterial virulence is not well understood.

*S. marcescens *produces two types of hemolysins: contact-dependent hemolysin and extracellular hemolysin [16]. The ShlA hemolysin has cytolytic and contact-dependent hemolytic activity, but little is known about the *S. marcescens *secreted hemolysin. The gene cassette responsible for the production and secretion of ShlA is *shl*AB, with *shl*A encoding the structural gene for hemolytic activity and *shl*B required for activation and secretion of ShlA in the presence of the cofactor phosphatidylethanolamine [17]. ShlA production is higher at 30°C than at 37°C [18].

In this study, we cloned an *S. marcescens *gene that produced hemolytic activity on human blood agar plates. The gene, designated *phl*A, encoded an extracellular PLA activity. We also showed that PhlA hydrolyzed phospholipid to lysophospholipid, which directly lysed human, horse, and sheep RBC and cultured cells.

## Methods

### Reagents

Taurocholic acid sodium salt hydrate and ethylenediamine-N,N'-diacetic acid (EDDA), L-α-phosphatidylcholine (PC) and L-α-phosphatidylethanolamine (PE) were purchased from Sigma. Cardiolipin (CL), L-3-phosphatidylinositol (PI) and sphingomyelin (SPM) and L-α-lysophosphatidylcholine (LPC) were purchased from Doosan Serdary Research Laboratories. Lecithin from egg yolk was purchased from Wako Chemicals. Phospholipase A2 derived from bovine pancreas was purchased from Sigma.

### Bacterial strains, plasmids, and media

*S. marcescens *niid 298 strain is one of our reference strains for serotyping, which is originally isolated from urine. *E. coli *K-12 DH5α and pBR322 were used for shotgun cloning. The pGEM-Easy vector (Promega) was used for cloning. pCold1 and pG-KJE8 (TaKaRa) were used as expression vectors in *E. coli *BL21 [F^-^, *ompT*, *hsdSB *(rB^- ^mB^-^), *gal*, *dcm*] (TaKaRa). Unless otherwise specified, bacteria were grown in Luria broth (LB). Antibiotics were added as required for the following final concentrations: ampicillin, 200 μg/ml; kanamycin, 100 μg/ml; and chloramphenicol, 20 μg/ml.

Peripheral human blood was obtained from healthy volunteers with their informed consent according to the guidelines laid down by the Ethics Committee of National Institute of Infectious Diseases. Horse and sheep blood were obtained from Kohjinbio. RBC were washed three times with phosphate-buffered saline (PBS) by centrifugation at 850 × g for 10 min. Washed RBC were resuspended in PBS to a final concentration of 2.5% (vol/vol) and used for hemolytic activity assays. Blood agar plates contained 12.5 g Bacto-tryptone, 5 g NaCl, 5% (v/v) RBC, 10 mM CaCl_2_, and 15 g agar (per liter), and the pH was adjusted to 7.2 [19]. PCY medium plates, which contained 10 g Bacto-tryptone, 5 g yeast extract, 5 g NaCl, 20 mM CaCl_2_, 5 g taurocholic acid, 15 g egg yolk lecithin, and 15 g agar (per liter), were used for determining phospholipase activity [14].

### Functional cloning

Shotgun cloning was used to identify hemolytic factors as follows. *S. marcescens *strain niid 298 genomic DNA was digested with *Sau3*AI, ligated into a pBR322 vector *Bam*HI site, and introduced into *E. coli *DH5α. Colonies that induced hemolysis on human blood agar were selected as candidate hemolysin clones. The inserted fragments were amplified by PCR and complete fragment sequences were determined using a 3130 Genetic Analyzer (Applied Biosystems). The *S. marcescens *nucleotide sequences determined in this work have been deposited in the DDBJ/EMBL/GenBank databases under the following accession numbers: AB505202 and AB505203 for the *phl*A and *phl*B genes of *S. marcescens *niid 298.

### Construction of mutant strains

A one-step inactivation method [19-21] was used to obtain *shlBA *and *phlAB *deletion mutants. For construction of a *shlBA *deletion mutant, PCR products were amplified from pKD4 [19] with primers ShlBA5' (5'-GGTTAACCTCATGGATTGGGCTGGCTGCCCCGGCGGCCTCTCATAGTGTAGGCTGGAGCTGCTTC-3') and ShlBA3' (5'-GCAAAACTCCACGCCTGCCGTCATGCTTCATGTCACTGTCAGCAACATATGAATATCCTCCTTAGT-3'), which flank the 5' and 3' termini of the *shlBA *gene with 45 bp homology, and electroporated into *S. marcescens *niid 298 carrying pKD46 [20]. For construction of a *phlAB *deletion mutant, primers PhlAB5' (5'-AGCGCCAGTAAGGCTATCGCCAGCGCCCGCCGCAAGCGACCCCCTCATATGAATATCCTCCTTAGT-3') and PhlAB3' (5'-TGCCTAAGAAAAAACCGCCTGTACAGGCGGTTTTTTTATGGGCGTCATATGAATATCCTCCTTAGT-3') were used. The correct mutation was verified by PCR using three different primer sets as described previously [20].

### Preparation of purified PhlA

The full-length *phlA *gene was obtained from *S. marcescens *niid 298 genomic DNA by PCR with primers phlA5' (5'-GAATTCCATATGAGTATGCCTTTAAGTTTTACCTCTG-3') and phlA3' (5'-GCTATCTAGATCAGGCATTGGCCTTCGCCTC-3'). The 5'- and 3'-termini of the PCR product were *Nde*I and *Xba*I restriction enzyme sites, respectively. The PCR fragment cleaved by these restriction enzymes was inserted into *Nde*I/*Xba*I-digested pCold1, which has a histidine tag site at the 5'-terminus, and used to transform *E. coli *DH5α. The resulting plasmid, pCold1-phlA, was introduced into *E. coli *BL21 harboring pG-KJE8 [22]. The transformant was used for expression of His-PhlA according to the manufacturer's instructions.

To purify the His-PhlA recombinant protein, cells were harvested, lysed by lysis buffer [50 mM NaH_2_PO_4_, 300 mM NaCl, 10 mM imidazole, and protease inhibitors (one tablet of inhibitor mixture (Complete, Roche)/25 ml)], incubated in a final concentration of 1 mg lysozyme/ml for 20 min on ice, and then disrupted by sonication. Cell debris was removed by centrifugation. The supernatant was analyzed by affinity chromatography using Ni-NTA agarose (Qiagen) under native conditions without a protease inhibitor. After dialysis against PBS, the purified protein was concentrated by Amicon Ultra-15 (MWCO = 30 K; Millipore). The protein concentration was determined using a BCA Protein Assay Kit (Pierce). We obtained approximately 1.9 mg purified recombinant protein (His-PhlA) from one liter of culture. The purified protein was analyzed by sodium dodecyl sulfate-polyacrylamide gel electrophoresis (SDS-PAGE) with Coomassie blue staining.

### Determination of hemolytic activity

The assay for contact-dependent hemolysis was performed as described previously [23]. Briefly, *S. marcescens *cells were cultured in LB containing EDDA (2 mM) at 30°C or 37°C and harvested at log phase. Bacteria (1.2 × 10^8 ^cells in 50 μl PBS) were mixed with 70 μl RBC and centrifuged (500 × g for 1 min). The mixture was incubated for 60 min at 30°C or 37°C with shaking. Hemoglobin released from lysed RBC was measured spectrophotometrically at 405 nm. Osmotic lysis of RBC in distilled water was taken as 100% hemolysis.

The hemolytic activity of purified PhlA in solution was measured as described previously [24,25], with the following modification. The RBC suspension containing 0.15 mg lecithin/ml, 0.06% taurocholic acid and 2 mM CaCl_2 _was incubated with His-PhlA at 37°C for 1 h. After centrifugation (500 × g for 10 min) the supernatant was assayed spectrophotometrically. RBC were not lysed by this low concentration of taurocholic acid.

### Detection of phospholipase A activity

Fluorogenic, BODIPY FL-labeled, phospholipase A substrates bis-BODIPY FL C_11_-PC, PED6, and PED-A1 (Invitrogen) were used to determine the specificities of PLA1 and PLA2. The bis-BODIPY FL C_11_-PC is glycerophosphocholine with BODIPY FL dye-labeled sn-1 and sn-2 acyl chains. PED-A1 and PED6 are glycerophosphoethanolamine with dye-labeled sn-1 and sn-2 acyl chains, respectively. The bis-BODIPY FL C_11_-PC was self-quenched, and PED-A1 and PED6 fluorescence was quenched by added dinitrophenol. Release of the fluorophores by acyl chain cleavage by either PLA1 or PLA2 results in increased fluorescence. Each substrate solution (45 nM) was prepared in 10 mM Tris-HCl (pH 8.0), 100 mM NaCl, and 10 mM CaCl_2 _[26]. A 90 μl sample of each substrate solution was incubated with various concentrations of enzymes (10 μl) in 96-well plates for 6 min, and fluorescence intensity was measured. The fluorescence background for each quenched substrate solution was determined without PhlA treatment. Fluorescence intensity was measured at 485 nm excitation and 530 nm emission using an Appliskan fluorescence microplate reader (Thermo Electron Corporation).

### Assay for free fatty acids from phospholipids

Non-esterified fatty acids (NEFA) released from phospholipids (PLs) were quantitated by an enzymatic colorimetric method using a NEFA-C kit (Wako chemical, Japan) [27]. Substrate solutions were prepared by dissolving 5 mg of various phospholipids in 1 ml of a solution of 2% taurocholic acid and 10 mM CaCl_2_. A 29 μl sample of each substrate solution was mixed with 1 μl His-PhlA and incubated at 37°C for 1 h. Background NEFA absorbance was estimated using non-His-PhlA treated substrates. NEFA concentrations were calculated from a calibration curve determined using oleic acid as a standard.

### Thin-layer chromatography

PC (0.65 mM) was incubated with 8.3 μM His-PhlA at 37°C for 1 h in the presence of 2% taurocholic acid and 10 mM CaCl_2_. The reaction was terminated by placing the samples on ice. The samples were dried with a SpeedVac concentrator, dissolved in 20 μl chloroform/methanol (2/1, vol/vol), applied to thin-layer chromatography plates (PE SILG; Whatman), and separated with chloroform/methanol/water (65/25/4, vol/vol/vol). LPC (1.0 mM) was analyzed on the same plate as a reference. Phospholipids on the plate were visualized with Dittmer-Lester reagent [28].

### Cell culture and cytolysis

HeLa and 5637 cells (derived from a human cervical cancer and bladder carcinoma, respectively) were grown in Dulbecco's Modified Eagle's Medium (DMEM) and 1640 RPMI medium, respectively, plus fetal calf serum (10% v/v) at 37°C in the presence of 5% CO_2_. At 24 h before the start of cytolysis experiments, 96-well culture plates were seeded with 1.0 × 10^4 ^cells per well. After washing with medium, the cells were incubated with various concentrations of His-PhlA in 100 μl lecithin solution (313 μg/ml lecithin, 0.125% taurocholic acid, and 2 mM CaCl_2 _in DMEM) at 37°C for 1 h. Cytolysis was measured as the amount of lactate dehydrogenase (LDH) released as determined with a CytoTox 96 Non-Radioactive Cytotoxicity Assay Kit (Promega) [29]. Complete (100%) cytolysis was determined by measuring LDH release after cell lysis with 1% Triton X-100.

## Results

### Identification of an *S. marcescens *hemolysin other than ShlA

**Figure 1:**
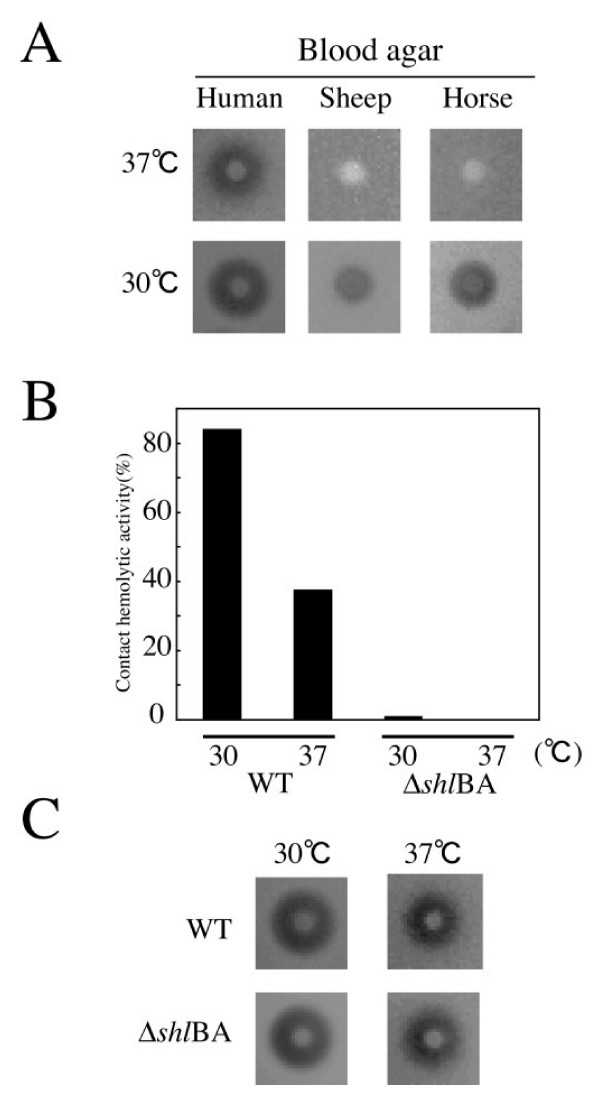
**Hemolytic activity of *S. marcescens***. (A) Hemolytic activity of *S. marcescens *strain niid 298 on several blood agars. Cells (1 × 10^6^) were cultured overnight, and then inoculated on various blood agars and incubated at 30°C or 37°C for 16 h. Clear zones indicated hemolysis. (B) Contact hemolysis assay for human RBC. Cells harvested in log phase were mixed with washed human RBC and incubated at 30°C or 37°C for 1 h with shaking. Released hemoglobin was measured spectrophotometrically as absorbance at 405 nm. Results are shown as percent lysis compared to complete lysis of RBC in distilled water. (C) Hemolytic activity of the *shlBA *deletion mutant on human blood agar. Experiments were performed as in (A).

*S. marcescens *niid 298 showed hemolytic activity visible as clear zones on human, sheep, and horse blood agar plates (Fig. 1A). The zones were larger for bacteria grown at 30°C than at 37°C. *S. marcescens *also showed contact-dependent hemolytic activity on human RBC, which was also greater for bacteria grown at 30°C than at 37°C (Fig. 1B).

Since ShlA is the only hemolysin that has been reported in *S. marcescens *[7], we constructed an *shlBA *deletion mutant. The mutant grown at both 30°C and 37°C lost its contact-dependent hemolytic activity (Fig. 1B), but retained hemolytic activity on human blood agar plates (Fig. 1C). These results indicated that *S. marcescens *had a hemolysin other than ShlA.

### Functional cloning of a novel hemolysin

To clone the *S. marcescens *hemolysin identified on human blood agar, we constructed a library of *S. marcescens *strain niid 298 DNA in *E. coli *DH5α. Approximately 13,000 transformants were examined for hemolytic activity on human blood agar plates and four hemolysin-positive clones were obtained. One clone showed hemolytic activity on human, sheep, and horse blood agar plates, but the other three clones showed activity only on human blood agar.

Sequence analysis of the inserts in the three clones with hemolytic activity only on human blood agar showed that all three had *phl*A and *phlB *genes with nucleotide similarity to *phlA *and *phlB *(94% and 94%, respectively) of *S. marcescens *MG1, which was originally classified as *S. liquefaciens *[13,15]. The *phlA *and *phlB *deduced amino acid sequences were similar to *Serratia *sp. MK1 PlaA and PlaB (81% and 73% identity) and *Y. enterocolitica *YplA and YplB (60% and 50% identity) [12,14]. PhlB has been suggested to be an inhibitor of PhlA inside the cell in which they are produced, thereby functioning to prevent PhlA activity until its release into the extracellular milieu [30]. Although there are no data about a PhlA hemolytic activity, since some other phospholipases have hemolytic activity, we investigated whether the *S. marcescens phlA *gene product might be a hemolysin.

### Hemolytic activity of *S. marcescens *PhlAB is on human blood agar

**Figure 2:**
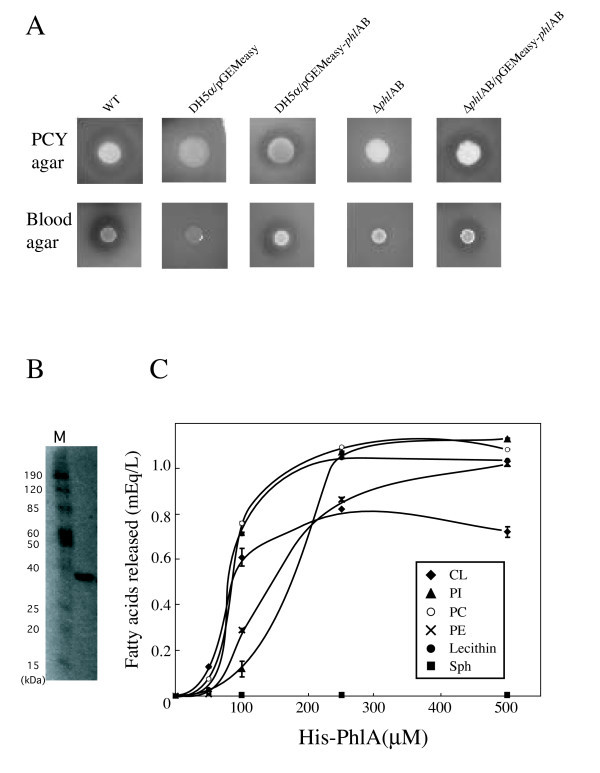
**Phospholipase and hemolytic activities of *S. marcescens *PhlA**. (A) Overnight cultures of wild-type strain *S. marcescens *niid 298, *E. coli *DH5αcells carrying pGEMeasy, *E. coli *DH5αcarrying pGEMeasy-*phlAB*, *S. marcescens *niid 298 *phlAB *deletion mutant, and *S. marcescens *niid 298 *phlAB *deletion mutant carrying pGEMeasy-*phlAB *(1 × 10^6 ^cells) were inoculated on blood agar plates and PCY agar plates and incubated at 37°C for 16 and 24 h, respectively. (B) Purified His-PhlA (1 μg) was separated by 12.5% SDS-PAGE, and then was stained with Coomassie blue. Protein standards were in lane M, with relative molecular masses (kDa) at the left. (C) Various phospholipids were mixed with His-PhlA and incubated at 37°C for 1 h. Free fatty acids (FFA) released from phospholipids were detected using a NEFA-C kit. The amount of FFA was determined from an oleic acid calibration curve. Values are averages ± SE of three independent experiments.

To confirm that *phlAB *had phospholipase and hemolytic activities, we constructed the *phlAB *expression vector pGEMeasy-*phlAB *and introduced it into *E. coli *DH5α. *E. coli *DH5α/pGEMeasy-*phlAB *showed a clear zone on PCY agar plates containing egg yolk lecithin as a substrate for phospholipase, in contrast to *E. coli *DH5α carrying an empty vector, indicating that PhlAB produced in *E. coli *DH5α/pGEMeasy-*phlAB *degraded phospholipids (Fig. 2A). In addition to phospholipase activity, *E. coli *DH5α/pGEMeasy-*phlAB *showed hemolytic activity on human blood agar plates (Fig. 2A).

We next constructed an *S. marcescens *niid 298 *phlAB *deletion mutant. The *S. marcescens *Δ*phl*AB mutant did not exhibit hemolytic activity on human blood agar plates or phospholipase activity on PCY agar plates (Fig. 2A). In contrast, the Δ*phl*AB mutant retained contact-dependent hemolytic activity against human RBC (data not shown). An *S. marcescens *Δ*phl*AB mutant carrying *phlAB *regained hemolytic and phospholipase activities (Fig. 2A), confirming that PhlAB had both activities.

### Characterization of recombinant His-PhlA protein

To investigate PhlA hemolytic and phospholipase activities, we purified a recombinant His-PhlA protein produced in *E. coli *(Fig. 2B). Purified His-PhlA had hemolytic activity human blood agar plates, but not on horse or sheep blood agar plates, and phospholipase activity on PCY agar plates (data not shown). These data indicated that PhlA had hemolytic and phospholipase activities, indicating that PhlB was not required for the PhlA activities.

We next studied the specificity of PhlA phospholipase. Phospholipase A (PLA) hydrolyzes the fatty acids of PLs at position *sn*-1 for phospholipase A1 (PLA1) and *sn*-2 for phospholipase A2 (PLA2), resulting in the release of free fatty acids and production of lysophospholipid (LPL). We measured free fatty acids after incubation of PhlA with various PLs [phosphatidylcholine (PC), cardiolipin (CL), L-3-phosphatidylinositol (PI), L-α-phosphatidylethanolamine (PE), and sphingomyelin (SPM)]. These experiments showed that PhlA cleaved ester bonds within PC, CL, PI, and PE and released fatty acids in a concentration-dependent manner, but did not hydrolyze SPM in our experimental conditions (Fig. 2C).

**Figure 3:**
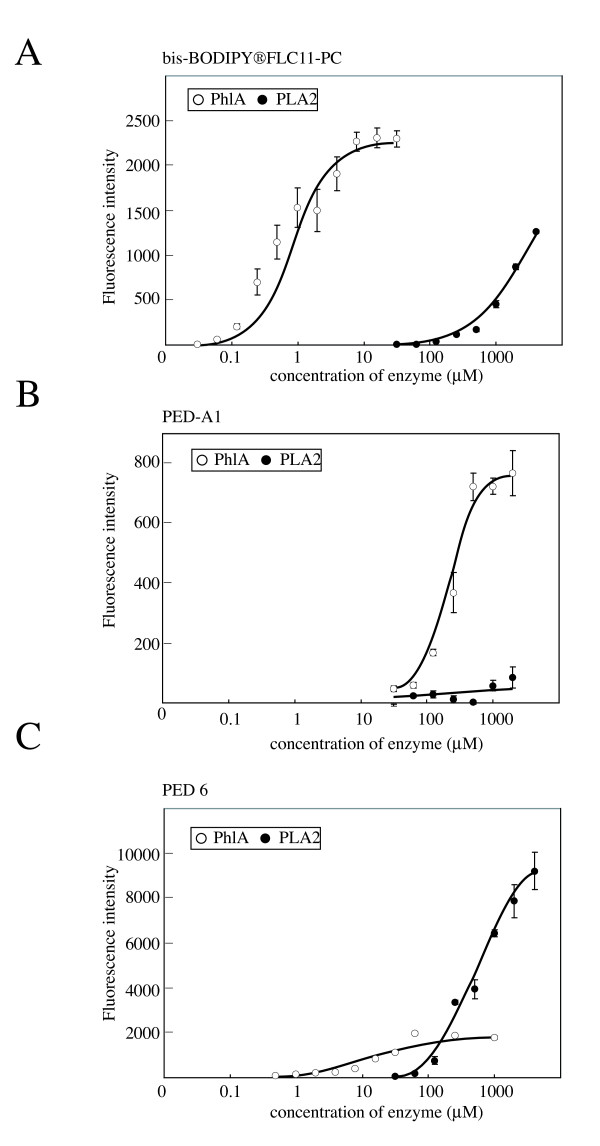
**PLA1 and PLA2 activities of PhlA. PhlA activity was evaluated in a fluorescence enhancement assay using the following PLA fluorescence substrates: (A) bis-BODIPYFLC11-PC, (B) PED-A1, and (C) PED6**. Fluorescence intensity was measured at 485 nm excitation and 530 nm emission using a fluorescence microplate reader (Appliskan; Thermo Electron Corporation). Open circles show His-PhlA; filled circles show PLA2 from bovine pancreas as a control. Values are averages ± SE from three independent experiments.

Previous reports have shown that some bacterial PLA2 enzymes have hemolytic activity [5,6,31]. However, there is little information on hemolysis caused by bacterial PLA1 enzymes. To confirm that *S. marcescens *PhlA had PLA1 activity, we tried to identify the site that is hydrolyzed by PhlA using fluorescent PLs as substrates [31,32]. As shown in Figure 3A, *S. marcescens *PhlA and bovine pancreatic PLA2 released fluorescent fatty acids from bis-BODIPY FLC11-PC, indicating that PhlA had phospholipase A activity (Fig. 3A). PhlA released fluorescent fatty acids from PED-A1 in a concentration-dependent manner whereas control PLA2 did not produce fluorescence (Fig. 3B), indicating that PhlA was able to cleave ester bonds at PL sn-1 sites. Using PED-6 as substrate, although fluorescence intensity increased after PhlA treatment, the maximum fluorescence was 6-fold lower than after PLA2 treatment (Fig. 3C). These results are in agreement with the proposal that His-PhlA has PLA1, but not PLA2, activity.

### PhlA requires phospholipids to express hemolytic activity

**Figure 4:**
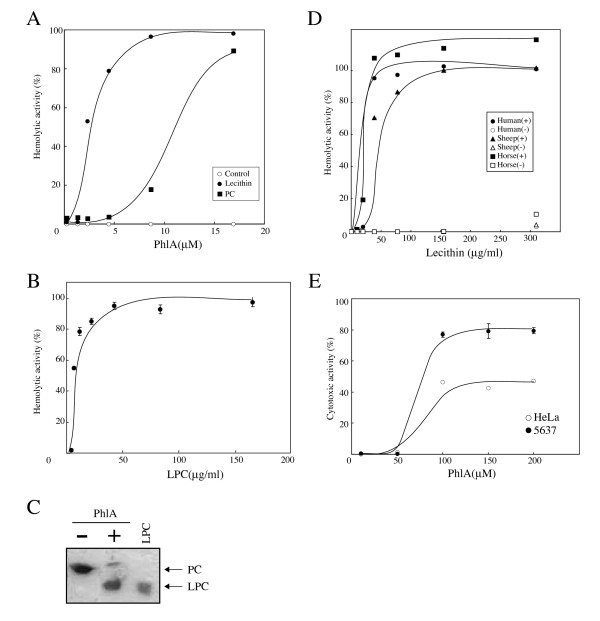
**Phospholipid requirements of PhlA hemolytic and cytotoxic activities**. (A) Human RBC were mixed with various concentrations of His-PhlA in the absence (open circles) or presence of lecithin (filled circles) or phosphatidylcholine (filled squares) and incubated at 37°C for 1 h. (B) Human RBC were mixed with various concentrations of lysophosphatidylcholine (LPC) and incubated at 37°C for 1 h. (C) Products of the reaction of PC with (+) or without (-) His-PhlA were analyzed by thin-layer chromatography. (D) Human (circles), sheep (triangles), and horse (squares) RBC were mixed with 8.3 μM PhlA (filled symbols) or no PhlA (open symbols) and incubated at 37°C for 1 h in the presence of various concentrations of lecithin with 2 mM CaCl_2_. (E) HeLa and 5637 cells were exposed to various concentrations of His-PhlA for 1 h in the presence of lecithin. His-PhlA cytotoxicity was evaluated with a CytoTox 96 Non-Radioactive Cytotoxicity Assay kit (Promega). Open and filled circles show HeLa and 5637 cells, respectively. Values are averages ± SE from three independent experiments. (A), (B), and (D) Results are expressed as percent lysis compared with lysis of RBC in distilled water, as in the contact hemolysis assay (Fig. 1).

The studies described above showed that PhlA had hemolytic and phospholipase A1 activities. To investigate PhlA activity on a range of target cells, we studied the activity of purified PhlA in a solution reaction system with different types of cells. Interestingly, in contrast to the results on blood agar plates, PhlA hemolytic activity on human RBC was not detected in solution reactions, even at a PhlA concentration as high as 18 mM (Fig. 4A). This result indicated that PhlA did not act directly as a hemolysin on RBC. It has been reported that several animal venoms containing PLA exhibit an indirect hemolytic activity in the presence of lecithin [23,24]. When egg yolk lecithin or PC was added to the PhlA solution reaction system, PhlA was observed to have indirect hemolytic activity on human RBC (Fig. 4A).

Lysophospholipid (LPL) is one of the products from PLs hydrolyzed by PLA1. Therefore, we investigated whether LPL could cause hemolysis of human RBC. Lysophosphatidylcholine (LPC) was found to have hemolytic activity on human RBC in the solution reaction system (Fig. 4B). Using thin-layer chromatography, LPC was found to be produced by incubation of PC with PhlA (Fig. 4C).

To determine the range of cells affected by PhlA, we examined various kinds of RBCs. As described above, PhlA lysed human RBC, but not horse or sheep RBC, on blood agar plates. However, all three types of RBC were lysed by PhlA in a lecithin-dependent manner in the solution reaction system (Fig. 4D). An explanation of these results may be that, in human blood agar plates, enough PL might be released from collapsed RBC during agar plate preparation to allow PhlA to produce LPL. When PL was added to sheep and horse blood agar plates, we clearly detected PhlA-dependent hemolytic activity on horse and sheep blood agar plates (data not shown).

Since PC required 6-fold more PhlA than lecithin for induction of 50% hemolysis (Fig. 4A), the egg yolk lecithin used in this study might have contained enough LPL for hemolysis. However, no hemolysis was induced by lecithin without PhlA treatment (Fig. 4D).

Taken together, these results indicated that PhlA phospholipase activity hydrolyzed PL and produced LPL. Since LPL is known to be a surfactant [33], it may have been the final effector leading to destabilization of the RBC membrane and hemolysis.

### Cytotoxicity of PhlA in the presence of phospholipid

We examined the cytotoxicity of PhlA using HeLa and 5637 cells. PhlA had cytotoxic activity against both HeLa and 5637 cells in the presence of lecithin (Fig. 4E). To investigate the cytolytic activity of late log phase *S. marcescens *culture supernatants, *S. marcescens *was grown at 37°C for 6 h in LB containing PL. Up to 48-fold dilutions of the *S. marcescens *culture supernatant induced cell death of both HeLa and 5637 cells, while supernatant of *S. marcescens *Δ*phlAB *cultured under the same conditions had no effect on HeLa or 5637 cells, indicating that PhlA was an extracellular secretion product (data not shown).

## Discussion

A wide range of pathogenic bacteria produce phospholipases, and the putative role of PLA in virulence has been studied in some of these pathogens. Outer membrane-associated PLAs (OMPLAs) were first identified in *E. coli *[34] and orthologs were subsequently reported in numerous gram-negative bacteria, including *H. pylori *(PldA) [9]. The OMPLAs have been well-characterized and are thought to enhance bacterial growth, colonization, and survival. In addition to modulation of the bacterial membrane, some OMPLAs were shown to have contact-dependent hemolytic/cytolytic activities [35]. Another group of PLAs (e.g., YplA [12], ExoU [36], PlaA [10], and SlaA [37]) is secreted from bacterial cells. Purified ExoU and SlaA [38,39] recombinant proteins do not show cytotoxic activity when added exogenously, and there is little information on the cytotoxicity of other secretory PLAs.

To our knowledge, ShlA is the only previously reported hemolysin from *S. marcescens*. Although, a Δ*shlAB *mutant showed hemolytic activity on blood agar plates, it did not exhibit contact-dependent hemolytic activity (Fig. 1C). Therefore, we performed functional cloning, which identified PhlA as an *S. marcescens *candidate hemolytic factor (Fig. 2A).

In the experiments reported here, we described the hemolytic and cytotoxic activities of *S. marcescens *PhlA. PhlA itself did not directly induce the destabilization of target cell membranes, but the LPL produced from PL by PhlA phospholipase activity showed hemolytic and cytolytic activities. Therefore, PhlA and ShlA have different hemolytic mechanisms. In addition, ShlA was expressed at lower temperature, but its expression decreased at 37°C [17]. In contrast, PhlA was expressed at 37°C, although its temperature regulation has not been elaborated. Also, PhlA hydrolyzed phosphoethanolamine (Fig. 3C), which is required for ShlA activity [16], implying that PhlA production could potentially regulate ShlA activity.

Tsubokura et al. [40] reported PL-dependent hemolytic activity in a *Y. enterocolitica *culture filtrate. Schmiel et al. [12] independently identified this hemolysin as a lecithin-dependent phospholipase A (YplA). However, there were no data on whether YplA also had cytotoxic activity in the presence of PL, similar to that reported here for *S. marcescens *PhlA.

PhlA cleaved the ester bond of PL at the *sn-1 *site, and produced fatty acids and LPL from several PLs; e.g., PC, PS, PE, and CL (Fig. 2C). LPL production by PL cleavage might explain why PL addition was required for PhlA hemolytic activity of (Fig. 4A), since LPL may act as a surfactant and induce hemolysis. We detected PhlA hemolytic activity on human blood agar, but not on sheep or horse blood agar (Fig. 1A). However, sheep and horse RBC were lysed with purified PhlA in the presence of PL. This difference may be explained if PLs are released from human RBCs during the preparation of blood agar, and then become substrates for added or secreted PhlA resulting in the production of LPL. In agreement with this possibility, we observed hemolysis around bacterial colonies by addition of egg yolk lecithin to sheep and horse blood agar plates (date not shown).

Our results on the mechanism of PhlA cytotoxic activity allowed us to quantitate cytotoxic activity in a liquid assay. Numerous reports have shown that bacterial phospholipases contribute to pathogenesis by directly hydrolyzing host membrane phospholipids and modulation of the host immune system via the production of lipid second messengers (5, 6, 31). Although PhlA did not produce direct cytotoxicity on cultured cells, the pathogenetic role of indirect cytotoxicity via LPL production should be investigated.

It has been reported that *Pseudomonas aeruginosa *ExoU inhibited neutrophil function in the lungs of infected mice [41] and group A *Streptococcus *(GAS) SlaA contributed to colonization of the upper respiratory tract [37]. Furthermore, a PhlA-like phospholipase, *Y. enterocolitica *YplA, has been shown to play a role in bacterial colonization of the intestinal tract and increasing the pathological changes resulting from the host inflammatory response in the mouse model [12]. The high degree of homology between YplA and PhlA suggests that PhlA may also play a role in *S. marcescens *colonization, since *S. marcescens *is thought to be a commensal in the intestinal tract where PLs are supplied by the host diet. The pathogenic role of PhlA remains to be elaborated.

## Conclusions

In this report, we have identified a hemolytic and cytotoxic factor in *S. marcescens *other than the previously reported ShlA. This new factor, PhlA, had phospholipase A1 activity. It is interesting to note that the hemolytic and cytotoxic activity of PhlA depend on the presence of phospholipids in addition to those in target cell membranes and seems to be mediated by the LPL product of the PhlA enzyme. Our results contribute to understanding the pathogenic role of microbial PLA.

## Abbreviations

CL: cardiolipin; EDDA: ethylenediamine-N,N'-diacetic acid; LPC: lysophosphatidylcholine; LPL: lysophospholipid; PC: phosphatidylcholine; PE: phosphatidylethanolamine; PI: phosphatidylinositol; PL: phospholipid; PLA: phospholipase A; RBC: red blood cells; SPM: sphingomyelin.

## Authors' contributions

KS carried out most of experimental works, and drafted the manuscript. SI performed the genetic studies. NK improved some of the experimental procedures. YG provided the draft genome sequence information. MO conceived the study and co-wrote the manuscript with HW. All authors have read and approved the final manuscript.

## References

[B1] Yu VL Serratia marcescens: historical perspective and clinical review The New England Journal of Medicine 1979 300 887 893 370597 10.1056/NEJM197904193001604

[B2] Hejazi A Falkiner FR Serratia marcescens J Med Microbiol 1997 46 903 912 10.1099/00222615-46-11-903 9368530

[B3] Hertle R The family of Serratia type pore forming toxins Curr Protein Pept Sci 2005 4 313 325 10.2174/1389203054546370 16101433

[B4] Palmer M The family of thiol-activated, cholesterol-binding cytolysins Toxicon 2001 39 1681 1689 10.1016/S0041-0101(01)00155-6 11595631

[B5] Shinoda S Matsuoka H Tsuchie T Miyoshi S Yamamoto S Taniguchi H Mizuguchi Y Purification and characterization of a lecithin-dependent haemolysin from Escherichia coli transformed by a Vibrio parahaemolyticus gene J Gen Microbiol 1991 137 2705 2711 1791426 10.1099/00221287-137-12-2705

[B6] Walker DH Feng HM Popov VL Rickettsial Phospholipase A2 as a pathogenic mechanism in a model of cell injury by typus and spotted fever group rickettsiae Am J Trop Med Hyg 2001 65 936 942 11792002 10.4269/ajtmh.2001.65.936

[B7] Hertle R Schwarz H Serratia marcescens internalization and replication in human bladder epithelial cells BMC Infect Dis 2004 4 16 10.1186/1471-2334-4-16 15189566 PMC441377

[B8] Sakurai J Nagahama M Oda M Clostridium perfringens alpha-toxin: characterization and mode of action J Biochem 2004 136 569 574 10.1093/jb/mvh161 15632295

[B9] Dorrell N Martino MC Stabler RA Ward SJ Zhang ZW McColm AA Farthing MJG Wren BW Characterization of Helicobacter pylori PldA, a phospholipase with a role in colonization of the gastric mucosa Gastroenterology 1999 117 1098 1104 10.1016/S0016-5085(99)70394-X 10535872

[B10] Flieger A Neumeister B Cianciotto N Characterization of the gene encoding the major secreted lysophospholipase A of Legionella pneumophila and its role in detoxification of lysophosphatidylcholine Infect Immun 2002 70 6094 6106 10.1128/IAI.70.11.6094-6106.2002 12379686 PMC130422

[B11] Grant KA Belandia IU Dekker N Richrdson PT Park SF Molecular characterization of pldA, the structural gene for a phospholipase A from Campylobacter coli, and its contribution to cell-associated hemolysis Infect Immun 1997 65 1172 1180 9119448 10.1128/iai.65.4.1172-1180.1997 PMC175114

[B12] Schmiel DH Wagar E Karamanou L Weeks D Miller VL Phospholipase A of Yersinia enterocolitica contributes to pathogenesis in a mouse model Infect Immun 1998 66 3941 3951 9673284 10.1128/iai.66.8.3941-3951.1998 PMC108459

[B13] Givskov M Olsen L Molin S Cloning and expression in Escherichia coli of the gene for extracellular phospholipase A1 from Serratia liquefaciens J Bacteriol 1988 170 5855 5862 3056919 10.1128/jb.170.12.5855-5862.1988 PMC211692

[B14] Song JK Kim MK Rhee JS Cloning and expression of the gene encoding phospholipase A1 from Serratia sp. MK1 in Escherichia coli J Biotechnol 1999 72 103 114 10.1016/S0168-1656(99)00096-6 10406101

[B15] Labbate M Zhu H Thung L Bandara R Larsen MR Willcox MD Givskov M Rice SA Kjelleberg S Quorum-sensing regulation of adhesion in Serratia marcescens MG1 is surface dependent J Bacteriol 2007 189 2702 2711 10.1128/JB.01582-06 17237163 PMC1855814

[B16] Goluszko P Nowacki MR Extracellular haemolytic activity of Serratia marcescens FEMS Microbiol Lett 1989 61 207 211 10.1111/j.1574-6968.1989.tb03580.x 2689279

[B17] Hertle R Brutsche S Groeger W Hobbie S Koch W Konninger U Braun V Specific phosphatidylethanolamine dependence of Serratia marcescens cytotoxin activity Mol Microbiol 1997 26 853 865 10.1046/j.1365-2958.1997.6031978.x 9426124

[B18] Poole K Braun V Influence of growth temperature and lipopolysaccharide on hemolytic activity of Serratia marcescens J Bacteriol 1988 170 5146 5152 3053645 10.1128/jb.170.11.5146-5152.1988 PMC211583

[B19] Saitoh T Iyoda S Yamamoto S Lu Y Shimuta K Ohnishi M Terajima J Watanabe H Transcription of the ehx enterohemolysin gene is positively regulated by GrlA, a global regulator encoded within the locus of enterocyte effacement in enterohemorrhagic Escherichia coli J Bacteriol 2008 190 4822 4830 10.1128/JB.00231-08 18487325 PMC2447015

[B20] Datsenko KA Wanner BL One-step inactivation of chromosomal genes in Escherichia coli K-12 using PCR products Proc Natl Acad Sci USA 2002 97 66400 66405 10.1073/pnas.120163297 PMC18686 10829079

[B21] Iyoda S Watanabe H Positive effects of multiple pch genes on expression of the locus of enterocyte effacement genes and adherence of enterohaemorrhagic Escherichia coli O157: H7 to HEp-2 cells Microbiology 2004 150 2357 2371 10.1099/mic.0.27100-0 15256577

[B22] Nishihara K Kanemori M Yanagi H Yura T Overexpression of trigger factor prevents aggregation of recombinant proteins in Escherichia coli Appl Environ Microbiol 2000 66 884 889 10.1128/AEM.66.3.884-889.2000 10698746 PMC91917

[B23] Braun V Günther H Neuss B Tautz C Hemolytic activity of Serratia marcescens Arch Microbiol 1985 141 371 376 10.1007/BF00428852 3893355

[B24] Gene JA Gomez M Gutierrez JM Cerdas L Neutralization of hyaluronidase and indirect hemolytic activities of Costa Rican snake venoms by a polyvalent antivenom Toxicon 1985 23 1015 1018 10.1016/0041-0101(85)90397-6 4095702

[B25] Al-Abdulla IH Sidki AM Landon J An indirect haemolytic assay for assessing antivenoms Toxicon 1991 29 1043 1046 10.1016/0041-0101(91)90087-8 1949062

[B26] Hatic SO II Picking WL Young BM Young GM Picking WD Purification and characterization of two active derivatives of recombinant YplA, a secreted phospholipase from Yersinia entercolitica Biochem Biophys Res Commun 2002 292 463 467 10.1006/bbrc.2002.6690 11906185

[B27] Kasurinen J Vanha-Perttula T An enzymatic colorimetric assay of calcium-dependent phospholipases A Anal Biochem 1987 164 96 101 10.1016/0003-2697(87)90373-3 3674375

[B28] Dittmer JC Lester RL A simple, specific spray for the detection of phospholipids on thin-layer chromatograms J Lipid Res 1964 15 126 127 14173318

[B29] Behl C Davis JB Lesley R Schubert D Hydrogen peroxide mediates amyloid beta protein toxicity Cell 1994 77 817 827 10.1016/0092-8674(94)90131-7 8004671

[B30] Givskov M Molin S Secretion of Serratia liquefaciens phospholipase from Escherichia coli Mol Microbiol 1993 8 229 42 10.1111/j.1365-2958.1993.tb01567.x 8316077

[B31] Sitkiewicz I Stockbauer KE Sitkiewicz JM Secreted bacterial phospholipase A2 enzymes: better living through phospholipolysis Trends Microbiol 2007 15 63 69 10.1016/j.tim.2006.12.003 17194592

[B32] Hendrickson HS Hendrickson EK Johnson ID Farber SA Intramolecularly quenched BODIPY-labeled phospholipid analogs in phospholipase A(2) and platelet-activating factor acetylhydrolase assays and in vivo fluorescence imaging Anal Biochem 1999 276 27 35 10.1006/abio.1999.4280 10585741

[B33] Silverman BA Weller PF Shin ML Effect of erythrocyte membrane modulation by lysolecithin on complement-mediated lysis J Immunol 1984 132 386 391 6690605

[B34] Scandella CJ Kornberg A A membrane-bound phospholipase A1 purified from Escherichia coli Biochemistry 1971 10 4447 4456 10.1021/bi00800a015 4946924

[B35] Istivan TS Coloe PJ Phospholipase A in Gram-negative bacteria and its role in pathogenesis Microbiology 2006 152 1263 1274 10.1099/mic.0.28609-0 16622044

[B36] Finck-Barbançon V Goranson J Zhu L Sawa T Wiener-Kronish JP Fleiszig SM Wu C Mende-Mueller L Frank DW ExoU expression by Pseudomonas aeruginosa correlates with acute cytotoxicity and epithelial injury Mol Microbiol 1997 25 547 557 10.1046/j.1365-2958.1997.4891851.x 9302017

[B37] Banks DJ Beres SB Musser JM The fundamental contribution of phages to GAS evolution, genome diversification and strain emergence Trends Microbiol 2002 10 515 521 10.1016/S0966-842X(02)02461-7 12419616

[B38] Phillips RM Six DA Dennis EA Ghosh P In vivo phospholipase activity of the Pseudomonas aeruginosa cytotoxin ExoU and protection of mammalian cells with phospholipase A2 inhibitors J Biol Chem 2003 278 41326 41332 10.1074/jbc.M302472200 12915403

[B39] Sitkiewicz I Nagiec MJ Sumby P Butler SD Cywes-Bentley C Musser JM Emergence of a bacterial clone with enhanced virulence by acquisition of a phage encoding a secreted phospholipase A2 Proc Natl Acad Sci USA 2006 103 16009 16014 10.1073/pnas.0607669103 17043230 PMC1635118

[B40] Tsubokura M Otsuki K Shimohira I Yamamoto H Production of indirect hemolysin by Yersinia enterocolitica and its properties Infect Immun 1979 25 939 942 500194 10.1128/iai.25.3.939-942.1979 PMC414537

[B41] Diaz MH Shaver CM King JD Musunuri S Kazzaz JA Hauser AR Pseudomonas aeruginosa induces localized immunosuppression during pneumonia Infect Immun 2008 76 4414 4421 10.1128/IAI.00012-08 18663007 PMC2546837

